# Estimation Model for Cotton Canopy Structure Parameters Based on Spectral Vegetation Index

**DOI:** 10.3390/life15010062

**Published:** 2025-01-07

**Authors:** Yaqin Qi, Xi Chen, Zhengchao Chen, Xin Zhang, Congju Shen, Yan Chen, Yuanying Peng, Bing Chen, Qiong Wang, Taijie Liu, Hao Zhang

**Affiliations:** 1 Aerospace Information Research Institute, Chinese Academy of Sciences, Beijing 100094, China; qiyq@aircas.ac.cn (Y.Q.); chenxi@aircas.ac.cn (X.C.); chenzc@aircas.ac.cn (Z.C.); 2 Research Institute, Xinjiang Academy Agricultural and Reclamation Science, Shihezi 832003, China; qiaqia0412@21cn.com (X.Z.); shencongju@163.com (C.S.); wangqionghope@126.com (Q.W.); 15299090691@163.com (T.L.); 3 The Oasis Key Laboratory of Ecological Agriculture of Xinjiang, Shihezi University, Shihezi 832003, China; yanzi@163.com; 4 College of Arts and Sciences, Lewis University, Romeoville, IL 60446, USA; pengyu@lewisu.edu

**Keywords:** spectral vegetation index, cotton canopy information, estimation model

## Abstract

The spectral vegetation indices derived from remote sensing data provide a detailed spectral analysis for assessing vegetation characteristics. This study investigated the relationship between cotton yield and canopy spectral indices to develop yield estimation models. Spectral reflectance data were collected at various growth stages using an ASD FieldSpec Pro VNIR 2500 spectrometer. Six prediction models were developed using spectral vegetation indices, including the Normalized Difference Vegetation Index (*NDVI*) and Ratio Vegetation Index (*RVI*), to estimate the Leaf Area Index (*LAI*) and above-ground biomass. For *LAI* estimation using the *NDVI*, the power function model (*y = 10.083x^11.298^*) demonstrated higher precision, with a multiple correlation coefficient of *R*^2^ = 0.8184 and the smallest root mean square error (*RMSE* = 0.3613). These results confirm the strong predictive capacity of *NDVI* for *LAI*, with the power function model offering the best estimation accuracy. In estimating above-ground biomass using *RVI*, the power function model of *y = 6.5218x^1.33917^* achieved the higher correlation (*R*^2^ = 0.8851) for fresh biomass with an *RMSE* of 0.1033, making it the most accurate. For dry biomass, the exponential function model (*y = 9.1565 × 10^−5^∙exp(1.1146x)*) was the most precise, achieving an *R*^2^ value of 0.8456 and the lowest *RMSE* value of 0.0076. These findings highlight the potential of spectral remote sensing for accurately predicting cotton canopy structural parameters and biomass weights. By integrating spectral analysis techniques with remote sensing, this research offers valuable insights for precision cotton planting and field management, enabling optimized agricultural practices and enhanced vegetation health monitoring.

## 1. Introduction

Over the past decades, advances in spectral remote sensing imaging have provided new approaches of monitoring vegetation canopy structure changes, predicting plant growth, and estimating crop yield and productivity [1,2,3,4,5,6] compared with broadband indices, spectral imaging, and non-imaging data technology, utilizing narrowband-based vegetation indices derived from canopy reflectance, providing a more accurate estimation of crop parameters [7,8,9]. Spectral imaging/Non-imaging remote sensing serves not only to enhance the recognition of crop and vegetation types but also to monitor crop growth, retrieve physiochemical characteristics, diagnose nutrition status, extract crop canopy information, and estimate agronomic parameters and chemical components [6,10,11]. Thus, spectral technology holds unique application potential in regional and global crop yield estimation, precision regulation of water and fertilizer usage, and monitoring of diseases and insect pests [12].

Vegetation canopy structural properties are critical variables in the exchange of energy, water, and CO_2_ between the atmosphere and the land surface [13]. Among the parameters describing the vertical profile of plant canopies, the Leaf Area Index (*LAI*) plays a pivotal role in plant ecological and biophysical processes [14,15]. It serves as a key indicator of crop canopy structure and growth, reflecting foliage cover, crop development, and yield potential [16,17,18]. Monitoring changes in *LAI* provides essential insights for optimizing cotton fertilization strategies [19,20].

The swift, accurate, and non-destructive estimation of cotton *LAI* is crucial for guiding crop management practices. Hyperspectral vegetation indices (*HVIs*) have been developed to estimate *LAI* by establishing statistical relationships between *HVIs* and measured *LAI* across various vegetation types. Among these indices, the Normalized Difference Vegetation Index (*NDVI*) stands out as the most widely recognized and utilized, serving as a cornerstone for estimating *LAI* variations [21]. Remote sensing technology enables timely, dynamic, and large-scale monitoring, making it an indispensable tool for tracking crop growth and development [11].

Cotton is a globally significant cash crop and a major contributor to the natural fiber supply. China, as one of the leading cotton producers, accounts for 25.6% of the world’s total cotton output [22]. Among its regions, Xinjiang stands out, contributing 87.3% to the national output and 22.3% to the global output in 2020. The canopy structure of cotton plants significantly influences their growth, making it a critical area of study. Spectral imaging and non-imaging remote sensing have been successfully applied to various aspects of cotton cultivation, including pest detection, irrigation management, and yield prediction [11]. In our research, we leverage spectral resolution techniques for the non-contact and non-destructive collection of field information related to cotton canopy structure [23]. By establishing relationships between canopy reflectance spectra and structural indices such as Leaf Area Index (*LAI*) and biomass, we aim to develop a spectral cognition retrieval model. This model will enhance remote sensing diagnostics for cotton, contributing to the advancement of precision cotton cultivation practices [24,25]. Remote sensing technology enables timely, dynamic, and macroscopic monitoring, making it an essential tool for tracking crop growth information. In recent years, numerous studies, both domestic and international, have utilized remote sensing technology to investigate crop biomass [14,20,26]. Spectral imaging and non-imaging remote sensing have proven effective not only in enhancing the identification of crop and vegetation types but also in monitoring crop growth, retrieving physiochemical characteristics, diagnosing nutritional status, extracting crop canopy information, and estimating agronomic parameters and chemical components [10]. Additionally, this technology shows unique application potential in areas such as yield estimation, precision regulation of water and fertilizer use, and monitoring of diseases and insect pests. As a pivotal tool for advancing precision agriculture, spectral remote sensing plays a crucial role in promoting sustainable agricultural development for the future [27].

In our research, we utilize spectral non-imaging data resolution techniques to enable rapid, efficient, non-contact, and non-destructive collection and processing of field information for the cotton canopy. This study focuses on investigating the relationship between canopy reflectance spectra and canopy structure indices with the aim of developing a spectral cognition retrieval model. This model incorporates key indices such as the Leaf Area Index (*LAI*) and biomass across various growth stages. The findings highlight the potential of this technique in advancing cotton remote sensing diagnosis, accelerating progress in remote sensing quantitative research, and providing a theoretical foundation for precision cotton cultivation.

## 2. Materials and Methods

The experiment was conducted at the Field Experimental Station of Shihezi University, Xinjing, China (44°20′ N, 86°3′ E). The region is characterized by a low mountain and hill terrain, with elevations ranging from 46 m to 114 m above sea level and slopes varying from 5° to 20°. The study area has a typical monsoon subtropical climate, with a mean annual temperature of 17.2 °C. The lowest monthly mean air temperature occurs in January at 4.7 °C, while the highest is recorded in July at 29.4 °C. The annual mean rainfall is 1422 mm, with the majority occurring between April and August. The annual mean relative humidity averages over 80%. The study site has loam soil with medium fertility, containing 1.29% organic matter, 33.8 mg·kg^−1^ available nitrogen, 84 mg·kg^−1^ available phosphorus, and 300 mg·kg^−1^ available potassium [28].

The main cotton varieties included Xinluzao 9, Xinluzao 10, Xinluzao 13, Xinluzao 19, Paotai 1, and Zhongmian 36. The experiment started in 2010. The date for sowing dibbling was 23 April, seedling transplantation was on 29 April, and topping was on 10 July. Field management practices including film drip irrigation, diseases and pest control, and herbicides application were consistent with those applied in larger-scale fields.

### 2.1. Measuring Methods

#### 2.1.1. Spectral Measuring Method

The cotton canopy spectra were measured using an ASD Field Spec Pro VNIR 2500 spectrometer (Analytical Spectral Devices, Boulder, CO, USA). The spectra range covered 350 to 2500 nm. The resolution for spectra within 350 to 1000 nm was 3 nm and was 10 nm between 1000 and 2500 nm. Measurements were conducted during sunny and cloudless mornings, specifically between 11:30 a.m. and 14:00 p.m., at various growth stages of cotton, including early budding, full budding, early blooming, full blooming, full-boll, and boll opening. The measurements were taken in sample fields selected for their representativeness, uniformity, and absence of diseases and insect pests in the cotton canopy. The field angle was fixed at 25°, with the detector head being positioned vertically downward. The distance between the detector head and the canopy was maintained at 100 cm, and each treatment involved measuring 10 to 15 curves, with the average value serving as the spectra reflectance for the specified small area. The scanning time for each spectra curve was set at 0.2 s. Whiteboard proofreading was performed both before and after the measurement process.

#### 2.1.2. Measuring of *LAI*

In alignment with the positions of the measured canopy spectra, samples were collected, and the American CI-110 digital plant canopy structure analyzer was employed for Leaf Area Index (*LAI*) measurements.

#### 2.1.3. Biomass Measurements

During the biomass measurement process, samples were collected, and their fresh weights were recorded according to organ type. The total fresh weight (g·m⁻^2^) for a 1 m^2^ area was then calculated. The samples were subsequently treated to deactivate enzymes by being placed in an oven at 105 °C for 30 min, followed by drying at a constant temperature of 80 °C. Dry biomass measurements were conducted when the difference between two consecutive measurements, taken one hour apart, was ≤5%.

### 2.2. Spectral Data Analysis and Technique Method

#### 2.2.1. Multivariate Statistical Analysis

As highlighted in previous studies [29], multivariate statistical analysis is a widely used method for investigating vegetation and crop spectral data. In this study, independent variables included spectral indices, such as original spectral reflectance, first- and higher-order derivatives, logarithmic transformations, vegetation indices, and transformations of reflectance reciprocals. These indices, specifically derived from spectral data, offer more accurate and relevant information for vegetation monitoring indices.

The dependent variables comprised biophysical and biochemical indices, such as *LAI*, biomass, and other vegetation metrics. These variables formed the basis for constructing multivariate regression models [30]. The analysis was divided into two phases: regression model development and accuracy evaluation. Data collected in 2009 were utilized for both model development and accuracy assessment.

#### 2.2.2. Spectral Vegetation Indices

Spectral vegetation indices are crucial for estimating various biophysical and biochemical properties of vegetation, such as *LAI*, vegetation coverage, biomass, photosynthetically active radiation absorption, chlorophyll content, and nutrient levels [31]. The continuous nature of spectral data allows for the development of spectral vegetation indices, which provide a higher level of precision for these estimations. For instance, the Normalized Difference Vegetation Index (*NDVI*) and Ratio Vegetation Index (*RVI*) at specific wavelengths (λ_0_) are calculated as follows:

(1) $$NDVI=\frac{\left(NIR-R\right)}{\left(NIR+R\right)}$$

(2) $$RVI=\frac{NIR}{R}$$

where: *NIR*: near-infrared reflectance (760–1056 nm), and R: red reflectance (680–780 nm).

## 3. Results

### 3.1. Research of NDVI in Retrieval of LAI

**Figure 1:**
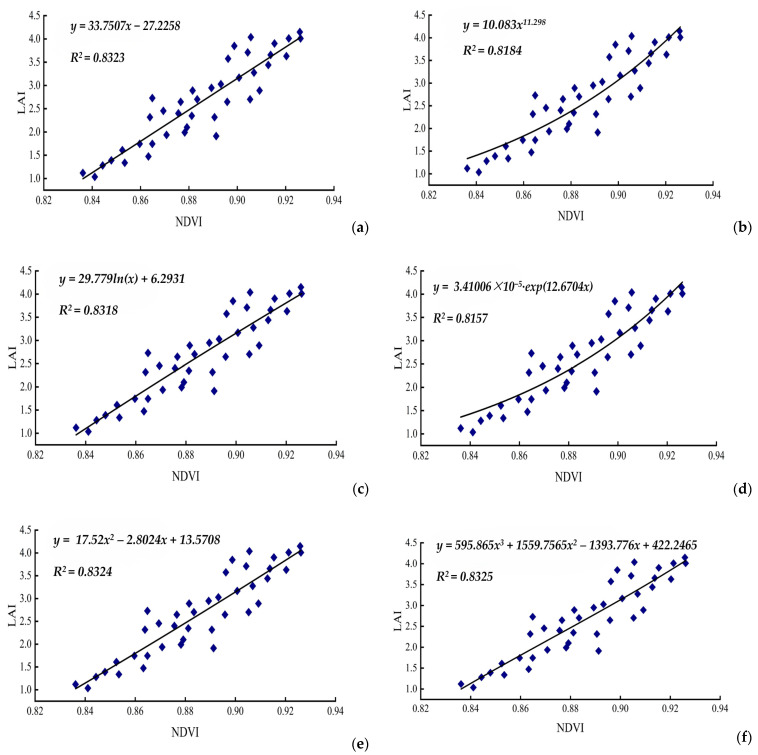
The correlation analysis between the *LAI* and *NDVI* of the cotton canopy. Note: (**a**). Simple linear function; (**b**). power function; (**c**). logarithmic function; (**d**). exponential function; (**e**). univariate quadratic function; (**f**). univariate cubic function.

As illustrated in Table 1 and Figure 1, the use of *NDVI* for predicting *LAI* across six models consistently yielded a high level of significance for the correlation coefficient (*R*) at α = 0.01. Among the models detailed in Table 1, the univariate cubic function demonstrated the highest multiple correlation coefficient (*R*^2^ = 0.8325) and a comparatively lower root mean square error (*RMSE*), indicating excellent estimation precision. This univariate model stands out as the most appropriate for spectral data analysis and effectively captures the mathematical relationship between *NDVI* and *LAI*.

Additionally, the univariate quadratic function model showed a similarly high multiple correlation coefficient (*R*^2^ = 0.8324). Conversely, the exponential function model exhibited the lowest correlation coefficient (*R*^2^ = 0.8157). The power function model of *y = 10.083x^11.298^* also demonstrated a high correlation coefficient and had the lowest *RMSE* value of 0.3613 among all models, signifying superior estimation accuracy and performance over the other models. This analysis confirms the practicality of employing *NDVI* to predict *LAI* in cotton, with both the univariate cubic and quadratic functions exhibiting strong predictive relationships.

For this study, we used composite *NDVI* values for *LAI* and reflectance measurements in the *NIR* and *R* bands from experimental cotton samples (*n* = 40). *NDVI* was considered the independent variable, while *LAI* served as the dependent variable, facilitating the development of six linear and non-linear predictive models.

### 3.2. Spectral Data Testing Model of RVI to Above-Ground Biomass

In our research, we developed a spectral data testing model utilizing the *RVI* to estimate above-ground dry biomass. The model incorporates the relationship between the *RVI* and the weight of above-ground dry biomass. The vegetation index (*VI*) serves as a crucial indicator in the estimation of biomass. Numerous research studies have substantiated the relationships between *VI* and *LAI*, as well as between *VI* and biomass.

**Figure 2:**
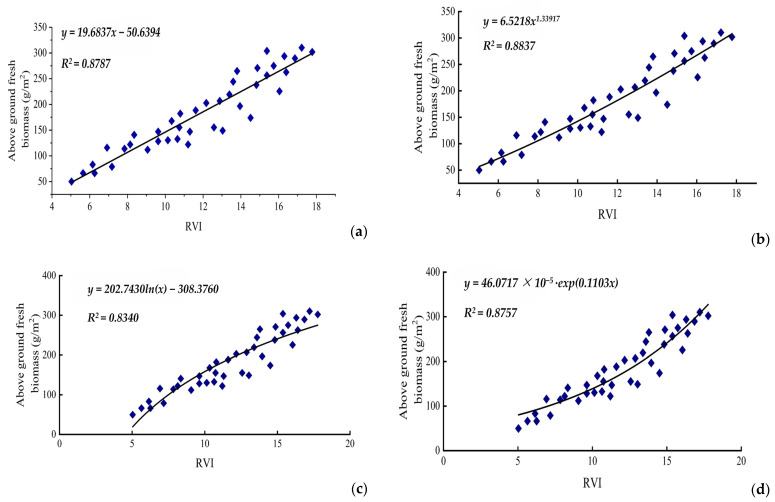

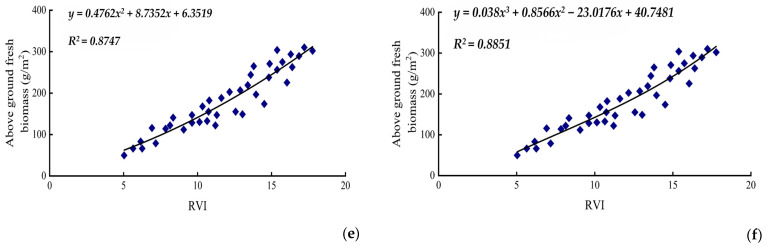
The correlation analysis between the fresh biomass and the *RVI* of cotton. Note: (**a**). Simple linear function; (**b**). power function; (**c**). logarithmic function; (**d**). exponential function; (**e**). univariate quadratic function; (**f**). univariate cubic function.

In our research, we used the *RVI* of experimental cotton samples to establish six prediction model equations for cotton unit area above-ground fresh biomass weights. As is shown in Figure 2, among the estimation models, the univariate cubic function model exhibits the highest multiple correlation coefficient (*R*^2^ = 0.8851), followed by the power exponent function model (*R*^2^ = 0.8837). The power function model also shows a high correlation coefficient with an *RMSE* of 0.1033 (Table 2), the lowest among the models.

### 3.3. Spectral Data Testing Model of RVI to Above-Ground Fresh Biomass

Table 2 and Figure 2 summarize the estimates of different model functions used to predict cotton fresh biomass based on the *RVI* (*n* = 40). Among the models evaluated, the power function model of *y = 6.5218x^1.33917^* showed the higher coefficient of determination (*R*^2^ = 0.8837) and the lowest root mean square error (*RMSE* = 0.1033), indicating superior accuracy in biomass estimation. The cubic model also demonstrated a strong performance with an *R*^2^ value of 0.8851 and an *RMSE* of 0.2985, matching the exponential model in terms of *RMSE*. The linear model yielded an *R*^2^ value of 0.8787 and an *RMSE* value of 0.3653, while the logarithmic mode had an *R*^2^ value of 0.8340 and an *RMSE* value of 0.1068. The exponential model produced an *R*^2^ value of 0.8757 and an *RMSE* value of 0.2985, and the quadratic model resulted in an *R*^2^ value of 0.8747 and an *RMSE* value of 0.4824. Overall, the power and cubic models were the most effective in estimating cotton fresh biomass, demonstrated by their high *R*^2^ and low *RMSE* values (Table 2 and Figure 2).

### 3.4. Spectral Data Testing Model of RVI to Above-Ground Dry Biomass

**Figure 3:**
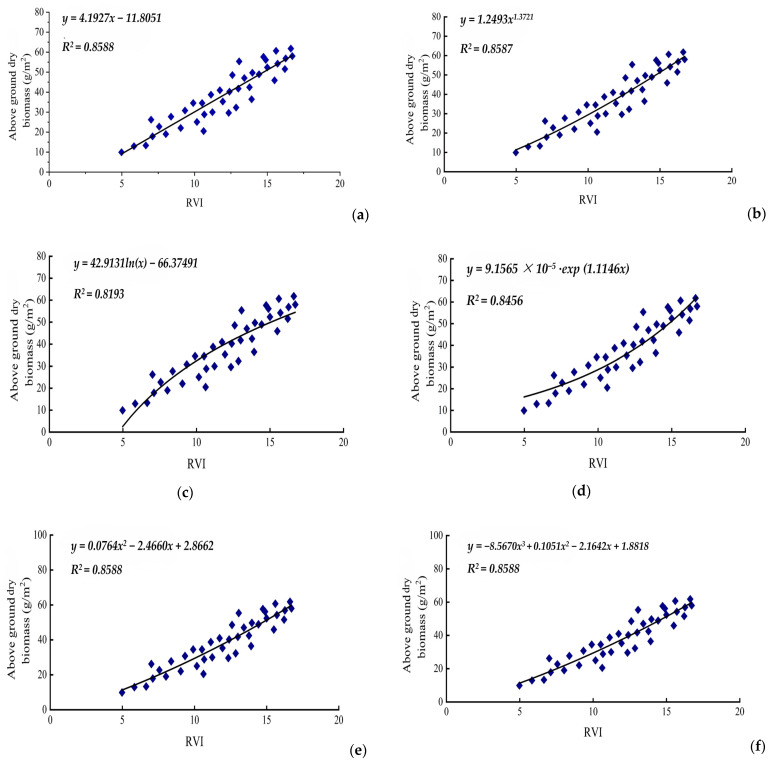
The correlation analysis between the dry biomass and the *RVI* of cotton. Note: (**a**). Simple linear function; (**b**). power function; (**c**). logarithmic function; (**d**). exponential function; (**e**). univariate quadratic function; (**f**). univariate cubic function.

In this study, we used the *RVI* from experimental cotton samples (*n* = 40) to develop six prediction model equations for estimating cotton unit area above-ground dry biomass. As shown in Figure 3, the cubic function model and univariate cubic function model exhibited the highest multiple correlation coefficient (*R*^2^ = 0.8588), closely followed by the power function model (*R*^2^ = 0.8587).

Table 3 and Figure 3 present the evaluation of the estimation models for cotton unit area above-ground dry biomass weights based on the *RVI*. The cubic function model and quadratic function model showed total root mean square errors (*RMSE*s) of 0.3924 and 0.3932, respectively, which were higher compared with other models. In contrast, the exponential function model of *y = 9.1565 × 10^−5^·exp(1.1146x)* demonstrated a high correlation coefficient and, notably, the lowest *RMSE* at 0.0076. This indicates that the exponential function model offers the highest estimation precision among the models evaluated, making it the most effective for predicting cotton unit area above-ground dry biomass.

## 4. Discussion

In our study, the primary focus was on leveraging spectral vegetation indices to establish remote sensing monitoring models for *LAI* and biomass. *LAI* is an important characteristic of plant canopies directly linked to energy acceptance, water exchange, and primary production in plant ecosystem. It is essential for monitoring changes in ecosystem carbon stocks and other ecosystem level fluxes [21,32].

The results presented in Table 1 underscore the robustness of utilizing *NDVI* as a predictor for *LAI* across various modeling approaches. The consistently high correlation coefficient (*R*) at α = 0.01 highlights the strong relationship between *NDVI* and *LAI*, emphasizing *NDVI*’s potential as a reliable proxy for estimating vegetation canopy structure [33]. Among the six models investigated, the power function model emerged as the most promising, exhibiting the higher multiple correlation coefficient (*R*^2^ = 0.8184) and the lowest root mean square error (*RMSE*). This indicates that the power function model provides highly precise estimations of *LAI* based on *NDVI* data, making it suitable for spectral applications and mathematical modeling of the *NDVI*–*LAI* relationship [34]. The high correlation coefficient observed for the univariate cubic function model (*R*^2^ = 0.8325) further supports the efficacy of non-linear modeling approaches in capturing the complexities of the *NDVI*–*LAI* relationship [34].

In order to estimate *LAI* in a large area (such as a regional scale), empirical relationships between *LAI* and spectral vegetation indices (*VIs*) retrieved from remotely sensed data were proposed. The regression models describing these relationships vary in mathematical forms (linear, exponential, power, inverse of exponential, etc.) [16,35]. In the present study, we found that the best-fit empirical relationships between *LAI* and *VIs* were represented by both the power and exponential function models, with coefficients of determination (*R*^2^) and *RMSE* values of 0.3613 and 0.3634, respectively. These results align with findings from other studies. For instance, exponential functions have been identified as the most suitable models for describing the empirical relationships between *LAI* and spectral indices in various crops, including corn, wheat, and soybean [16,36].

In our study, we used composite *NDVI* values, alongside *NIR* and *R* reflectance measurements, from experimental cotton samples to develop six predictive models for *LAI*, treating *NDVI* as the independent variable and *LAI* as the dependent variable. The high correlation coefficients across the models, as shown in Figure 1, validate the strength of the *NDVI*–*LAI* relationship and highlight the potential of *NDVI* as a valuable tool in agricultural and ecological research.

All six models achieved statistical significance at α = 0.01, with the power function model showing higher performance, demonstrated by an *R*^2^ of 0.8184 and an *RMSE* of 0.3613, making it particularly suitable for spectral analyses and modeling *NDVI*–*LAI* relationships. These results align with prior research findings [37]. Notably, all models showed correlation coefficients exceeding 0.8. The exponential function model ranked second in performance.

Comparing models for predicting above-ground fresh biomass using *NDVI* with those for dry biomass revealed that the former models demonstrated superior correlation. Among these, the power function model (*y = 6.5218x^1.33917^*) was the most effective, (*RMSE* = 0.1033). This indicates that using the power function model provides greater precision in estimating biomass in cotton, supporting its application for higher estimation accuracy in cotton yield assessments. This finding regarding a stronger exponential relation between vegetation index (*VI*) and biomass is consistent with results obtained by previous researchers [38]. Most investigations characterize the relationship between vegetation and biomass as either linearly correlated or exponentially correlated [39,40].

In this study, we developed a remote sensing model using the *RVI* to estimate above-ground dry biomass, emphasizing the role of vegetation indices (*VIs*) as vital indicators for biomass estimation. Prior research has shown correlations between *VI* and *LAI*, as well as between *VI* and biomass, often characterized as either linear or exponential [36]. Our focus was on applying the *RVI* from experimental cotton samples to derive six prediction models for estimating above-ground fresh biomass weights per unit area. The cubic function model had the highest multiple correlation coefficient (*R*^2^ = 0.8851) among these models. However, its root mean square error (*RMSE*) of 0.2985 was higher than that of other models. In contrast, the exponential function model demonstrated a high correlation coefficient and the lowest *RMSE* at 0.2895, indicating the best estimation accuracy. These results align with previous research findings [41]. Although still in its early stages, the use of spectral data in cotton research in Xinjiang presents significant potential for a wide range of future applications. There is considerable opportunity for the further development of models that leverage remote sensing and vegetation indices (*VIs*) for comprehensive monitoring of cotton growth. This could include assessing the nutritional status of cotton plants, guiding irrigation practices to optimize water use, enhancing cotton management and fertilization strategies, forecasting diseases and pest outbreaks, and enabling rapid predictions of cotton production and area estimates.

The application of *VIs* to estimate crop biomass is recognized as an effective and accessible method, especially during various stages of plant growth and development [2,42]. Prior research has highlighted variations in the estimation of crop biophysical parameters using *VIs* across different growth stages [42]. For instance, different *VIs* have been shown to be more or less effective for maize biomass estimation depending on the growth stage [42]. These differences are primarily attributed to the potential saturation of spectral indices following canopy closure and soil influences during the early stages of growth [43]. In this study, spectral-derived *VIs* were utilized to estimate cotton biomass at different growth stages, including early budding, full budding, early blooming, full blooming, full-boll, and boll opening. It was observed that the *LAI* and *RVI* varied across these stages, suggesting that plant growth stages should be a critical consideration when using canopy structure indices for biomass estimation. Integrating spectral *VIs* data at different growth stages into traditional *VIs*-based models could enhance the accuracy of biomass estimation, as demonstrated in studies on rice yield predictions [43].

## 5. Conclusions

In this study, six mathematical models—simple linear regression, power regression, logarithmic regression, exponential regression, quadratic regression, and cubic regression—were evaluated to estimate cotton biomass. Our analysis revealed that the exponential regression model was the most effective for estimating above-ground biomass, aligning with previous research that demonstrated that non-linear models generally offer higher prediction accuracy for biomass estimation compared with linear models [42]. Furthermore, studies involving multiple crops, including cotton, potato, soybeans, and corn, have found that non-linear models, particularly exponential functions, were most often the best fit for describing the relationship between crop biophysical variables and spectral indices [6].

This study focused on remote sensing techniques for monitoring cotton canopy growth. By conducting regression analyses of physical and spectral parameters, we developed mathematical models that successfully predicted cotton *LAI* and biomass at various growth stages. The findings confirm that remote sensing models based on spectral variables are effective for monitoring the dynamic changes in *LAI* and biomass throughout cotton growth [31]. These results provide a robust scientific foundation for future advancements in crop growth monitoring. The research also highlights the potential of spectral data as a promising tool for future applications, both from the ground and in aerospace platforms. Leveraging insights gained from these approaches can enhance vegetation monitoring capabilities, offering transformative opportunities for cotton research and agricultural practices in Xinjiang.

Study limitations and perspectives: We acknowledge the limitations of this study, particularly the absence of a multivariate statistical analysis involving vegetation indices and machine learning regression methods. A machine learning regression model was applied for prediction, utilizing a leave-one-out cross-validation (LOOCV) [44] approach to ensure robust results, given the small sample size. While our work demonstrates the utility of regression models for biomass estimation, incorporating machine learning approaches and leave-one-out cross-validation would likely provide more robust predictions, particularly given the limited sample size in this study. Future research should explore these methodologies to enhance the reliability and applicability of spectral data for *LAI* and biomass prediction. To address this, future studies should focus on calculating vegetation indices and leveraging their potential for more accurate predictions using advanced statistical and machine learning techniques. By addressing these limitations, our findings pave the way for further exploration of remote sensing techniques in agriculture. Future research should aim to integrate spectral imaging with machine learning algorithms to unlock its full potential in crop monitoring and management.

## Figures and Tables

**Table 1:** Estimates of cotton *LAI* model function base on *NDVI* (*n* = 40).

	Model Function	Regression Function	*R* ^2^	*RMSE*
1	*y = a + bx*	*y = 33.7507x − 27.2258*	0.8323	0.3967
2	*y = ax^b^*	*y = 10.083x^11.298^*	0.8184	0.3613
3	*y = a + bln(x)*	*y = 29.779ln(x) + 6.2931*	0.8318	0.3965
4	*y = a∙exp(bx)*	*y = 3.41006 × 10^−5^∙exp(12.6704x)*	0.8157	0.3634
5	*y = ax^2^ + bx + c*	*y = 17.52x^2^ – 2.8024x + 13.5708*	0.8324	0.4527
6	*y = ax^3^ + bx^2^ + cx + d*	*y = 595,865x^3^ + 1559.7565x^2^ – 1393.776x + 422.2465*	0.8325	0.4499

**Table 2:** Estimates of model function for cotton fresh biomass base on the *RVI* (*n* = 40).

	Model Function	Regression Function	*R* ^2^	*RMSE*
1	*y = a + bx*	*y = 19.6837x − 50.6394*	0.8787	0.3653
2	*y = ax^b^*	*y = 6.5218x^1.33917^*	0.8837	0.1033
3	*y = a + bln(x)*	*y = 202.7430ln(x) − 308.3760*	0.8340	0.1068
4	*y = a∙exp(bx)*	*y = 46.0717 × 10^−5^∙exp(0.1103x)*	0.8757	0.2985
5	*y = ax^2^ + bx + c*	*y = 0.4762x^2^ + 8.7352x + 6.3519*	0.8747	0.4824
6	*y = ax^3^ + bx^2^ + cx + d*	*y = 0.038x^3^ + 0.8566x^2^ − 23.0176x + 40.7481*	0.8851	0.2985

**Table 3:** Estimates of model function for cotton dry biomass base on the RVI (*n* = 40).

	Model Function	Regression Function	*R* ^2^	*RMSE*
1	*y = a + bx*	*y = 4.1927x − 11.8051*	0.8558	0.4473
2	*y = a x^b^*	*y = 1.2493x^1.3721^*	0.8587	0.1177
3	*y = a + b ln(x)*	*y = 42.9131ln(x) − 66.3749*	0.8193	0.3117
4	*y = a∙ exp(bx)*	*y = 9.1565 × 10^−5^∙exp(1.1146x)*	0.8456	0.0076
5	*y = ax^2^ + bx + c*	*y = 0.0764x^2^ − 2.4660x + 2.8662*	0.8588	0.3932
6	*y = ax^3^ + bx^2^ + cx + d*	*y = −8.5670x^3^ + 0.1051x^2^ − 2.1642x + 1.8818*	0.8588	0.3924

## Data Availability

The original contributions presented in this study are included in the article. Further inquiries can be directed to the corresponding authors.
